# Migrants Are Underrepresented in Mental Health and Rehabilitation Services—Survey and Register-Based Findings of Russian, Somali, and Kurdish Origin Adults in Finland

**DOI:** 10.3390/ijerph17176223

**Published:** 2020-08-27

**Authors:** Anu E. Castaneda, Katja Çilenti, Shadia Rask, Eero Lilja, Natalia Skogberg, Hannamaria Kuusio, Essi Salama, Jari Lahti, Marko Elovainio, Jaana Suvisaari, Seppo Koskinen, Päivikki Koponen

**Affiliations:** 1 Finnish Institute for Health and Welfare, 00271 Helsinki, Finland; katja.cilenti@thl.fi (K.Ç.); shadia.rask@thl.fi (S.R.); eero.lilja@thl.fi (E.L.); natalia.skogberg@thl.fi (N.S.); hannamaria.kuusio@thl.fi (H.K.); marko.elovainio@thl.fi (M.E.); jaana.suvisaari@thl.fi (J.S.); seppo.koskinen@thl.fi (S.K.); paivikki.koponen@thl.fi (P.K.); 2 Department of Psychology and Logopedics, University of Helsinki, 00014 Helsinki, Finland; jari.lahti@helsinki.fi; 3 Doctoral Programme in Clinical Research, Faculty of Medicine, University of Turku, FI-20014 Turku, Finland & Child Psychiatry, Turku University Hospital, 20521 Turku, Finland; essala@utu.fi

**Keywords:** migrant, mental health, service use, rehabilitation, psychiatry, population-based

## Abstract

Mounting evidence suggests that migration background increases the risk of mental ill health, but that problems exist in accessing healthcare services in people of migrant origin. The present study uses a combination of register- and survey-based data to examine mental health-related health service use in three migrant origin populations as well as the correspondence between the need and use of services. The data are from the Finnish Migrant Health and Wellbeing Study (Maamu), a comprehensive cross-sectional interview and a health examination survey. A random sample consisted of 5909 working-aged adults of Russian, Somali, and Kurdish origin of which 3000 were invited to participate in the survey and the rest were drawn for a register-based approach. Some of the mental health services, based on registers, were more prevalent in the Kurdish origin group in comparison with the general population and less prevalent in the Russian and Somali origin groups. All the migrant origin groups were underrepresented in rehabilitation services. When affective symptoms were taken into account, all the migrant origin groups were underrepresented in all of the services. This calls for actions to promote mental health, diminish the barriers to access services, and improve the service paths for migrants.

## 1. Introduction

Mental disorders are a major public health problem and have been estimated to constitute 10.4% of the global burden of disease [1]. Mounting evidence demonstrates that compared to the population in the country of settlement, migration background increases the risk of mental ill health [2,3,4]. Nevertheless, people of migrant origin face various barriers to accessing healthcare [5,6,7], although it is also important to recognize the heterogeneity between and among different migrant groups by country of origin and reason for migration [8]. Refugees and asylum seekers in particular have been shown to be at a greater risk of poor mental health [3,9,10]. Yet, despite the high prevalence of post-traumatic stress disorder (PTSD) amongst persons of refugee or asylum-seeking backgrounds, help-seeking behavior for psychological problems has consistently been found to be low [5,11,12].

According to previous studies, accessibility of healthcare services depends on a range of factors, related to e.g., the health system in the given country as well as to individual characteristics of persons seeking care [13]. Key barriers to seeking help for mental health problems include structural barriers (e.g., unstable housing), cultural barriers (e.g., mental health stigma), and barriers specific to refugees and asylum seekers (e.g., visa status and restricted access to health services) [5]. Using cross-sectional data from the European Statistics on Income and Living Conditions 2012, Guidi et al. [14] found that the prevalence of unmet health service needs was higher in women of foreign background when adjusting for age and health status, but no longer after adjusting for socioeconomic position. Discrimination is also one of the key components that may affect mental health [15,16] by internalized otherness as well as help-seeking behavior, of which both mechanisms should be studied more.

Rask et al. [17] demonstrated in a survey setting a high prevalence of affective symptoms among Russian origin women (24%) and Kurdish origin men (23%) and women (49%) in comparison to general population men (9%) and women (10%) in Finland. Moreover, a Finnish study by Castaneda et al. [18] found a high prevalence of potentially traumatic experiences in the former home country among persons of Somali and Kurdish origin. A register-based cohort study from Finland demonstrated that while the incidence and prevalence of mental disorders were overall lower among migrants compared with the native Finnish population, large risk differences emerged when examined by migrant origin and mental disorder group [19]. In addition, a recent register-based study in Finland showed less mental health service use among migrants than native Finnish controls, and that migrants from Eastern Europe, Middle East, and Africa have the highest risk of receiving low-intensity treatment [20]. Based on these separate studies, there appears to be a discrepancy between the observed high prevalence of mental health problems and the infrequent use of mental health services among people of migrant origin in Finland. These studies also point out the limitations of register-based studies in the evaluation of illness prevalence, since only persons in the service systems end up in registers. No previous studies have simultaneously examined the use of mental health services with respect to the need for these services. This would be of great interest and importance, since by using simultaneously register- and survey-based data, mental health-related health service use by need can be evaluated.

Ensuring adequate and equal access to health services should be a key objective for the European governments [13], including Finland. The UCL–Lancet Commission on Migration and Health has called for universal and equitable access to health services for all, including migrants [2]. Knowledge of the health of specific population groups is needed for developing and improving health services and individual clinical care [21,22]. Furthermore, understanding the social epidemiology of unmet need is needed to identify populations at risk of not receiving adequate mental health care [23].

This study examined in a setting using both register- and survey-based data (1) the mental health-related use of health services in three migrant origin populations in comparison to the general population, (2) the mental health-related use of health services among those in mental health-related need of services in the migrant origin groups in comparison to the general population, and (3) the difference between the migrant origin groups and the general population in reporting in survey mental health-related use or need of health services among those who according to the register data are receiving mental health-related health services. We hypothesized that (1) people of migrant origin are overrepresented in some services, measured with indicators such as having diagnoses, medications, and mental health-related sick leaves, but underrepresented in other more intensive services (such as rehabilitation services), (2) among the people who are in need of mental health treatment based on having affective symptoms, people of migrant origin are underrepresented in the services, and (3) survey-based information on service use and need of services is underreported more in the migrant origin groups compared to the general population.

## 2. Materials and Methods

### 2.1. Study Context in Finland

Finland is a Nordic welfare state characterized by a universal right to social welfare and healthcare services. Service delivery is mainly public, arranged and funded by municipalities for their residents. A much smaller sector of private providers and non-governmental organizations complement the system. The Social Insurance Institution of Finland (Kela) is a government agency that provides coverage for sickness-related expenses such as medical, medicine, and rehabilitation costs for all permanent residents of Finland through the National Health Insurance scheme. An entitlement to reimbursement of medicine expenses at a special rate can be granted on medical grounds based on a medical certificate issued by a doctor.

People with foreign background constitute approximately eight percent (423,494 persons at the end of 2019) of the total population in Finland [24]. The largest groups with migrant background in Finland are people from Russia or the former Soviet Union, followed by people from Estonia, Iraq, Somalia, the former Yugoslavia, China, and Vietnam. Looking at the reasons for immigration in 2019, 27% of the first residence permits consisted of third country nationals arriving on the grounds of family ties [25]. Approximately 25% of the permits were granted on the grounds of employment, 23% were registrations of EU-citizens, and 14% were residence permits for studying. Ten percent of the permits were granted on the basis of international protection or to resettled refugees.

### 2.2. Study Design, Procedure, and Participants

The data are from the Finnish Migrant Health and Wellbeing Study (Maamu) [26], a comprehensive cross-sectional interview and health examination survey conducted in Finland between 2010 and 2012 by the Finnish Institute for Health and Welfare (THL). The original study sample was randomly selected from the National Population Register. Six large cities were chosen to represent cities with a high proportion of people of migrant origin (Helsinki, Espoo, Vantaa, Turku, Tampere, and Vaasa). The random sample in these cities consisted of 3000 adults aged between 18 and 64 years of Russian, Somali, or Kurdish origin (1000 persons per each ethnic group). The number of the groups studied was limited to three due to financial and practical reasons (e.g., translating the survey measurements, coordinating the fieldwork of bilingual study personnel). The three groups were selected to represent different kinds of large foreign-origin groups in Finland. Russian or former Soviet Union (FSU) origin persons constituted the largest foreign-born group, Somali origin persons were the fourth largest group and the largest group with a refugee background and of Muslim faith, and Kurdish-speaking persons from Iraq or Iran were also among the largest groups, with Iraqi and Iranian refugees being among the largest groups of quota refugees accepted to Finland in the recent years. The inclusion criteria for persons of Russian origin were birthplace in Russia or FSU and native language Russian or Finnish, for persons of Somali origin birthplace in Somalia, and for persons of Kurdish origin birthplace in Iran or Iraq and native language Kurdish. The inclusion criteria also included residence in Finland for at least one year. Persons still living in reception centers failed to meet the inclusion criteria for having no permanent residency in any municipality, yet.

The sampled persons were invited to a comprehensive face-to-face interview (approx. 1.5 h) and a standardized health examination (approx. 1 h), with no fixed order. The structured interview included questions on socio-economic status and migration background, health, lifestyle, social wellbeing, safety, and health service use. The health examination included various measurements of health, functional capacity, and different kinds of symptoms. A short interview was offered to those refusing to participate in the full interview. Several health registers were drawn regarding the sample and linked with the survey data when possible. The fieldwork was conducted by trained bilingual personnel of Russian, Somali, and Kurdish origin. The participation rate in at least one part of the study (full interview and/or health examination and/or short interview) was 70.2% (*n* = 702) for Russian origin group, 51.2% (*n* = 512) for Somali origin group, and 63.2% (*n* = 632) for Kurdish origin group. A more detailed description of the study methodology can be found elsewhere [26].

An additional sample (Russian origin *n* = 998; Somali origin *n* = 963; Kurdish origin *n* = 948) was drawn from the National Population Register to be used in register-based analyses and for future follow-up studies. Thus, persons in the additional sample were not invited to participate in the interview or health examination but the additional sample is used in the present study regarding the register-based variables.

### 2.3. Comparison Group of the General Population

The comparison group of the general population in Finland was derived from the national population-based Health 2011 Survey [27]. The subsample included all sampled persons within the same age range and living in the same municipalities as in the Maamu Study (*n* = 2275). Of this sample, 70% participated in at least one part of the study (*n* = 1582). The Health 2011 Survey followed a comparable study protocol as the Maamu Study.

### 2.4. Register-Based Data and Measurements

Demographic characteristics of the study sample (gender, age, country of origin, native language) were obtained from the National Population Register.

Information on visits to a hospital inpatient or outpatient care in specialized health services was obtained from the Hospital Discharge Register, maintained by THL. The registers of Social Insurance Institution were used to obtain information on the purchase of prescribed psychotropic medication, entitlement to reimbursement of psychotropic medicine expenses at a special rate, sick leaves for more than ten days, and use of rehabilitation services.

For the purpose of the present study, altogether eight register-based variables were formed: (1) having at least one visit to hospital inpatient or outpatient care in specialized services with a psychiatric diagnosis (ICD-10 diagnosis codes F00–F99 [28]; 2009–2012), (2) having at least one visit to hospital inpatient or outpatient care in psychiatry (2009–2012), (3) purchase of prescribed psychotropic medication (Anatomical Therapeutic Chemical ATC—codes N05 and N06 [29]; 2009–2011), (4) entitlement to reimbursement of psychotropic medicine expenses at a special rate (112 severe psychoses and other severe psychiatric disorders [30]; 2009–2011), (5) sick leaves for more than ten days due to psychiatric diagnosis (ICD-10 diagnosis codes F00–F99 [28]; 2009–2011), (6) use of psychotherapeutic rehabilitation ([31]; 2009–2012), (7) a combination variable of any of the above mentioned register-based records on mental health-related use of health services (variables 1–6), and (8) use of any rehabilitation (i.e., general rehabilitation, including psychotherapeutic rehabilitation [31]; 2009–2012). The last variable of general rehabilitation was added to variables of interest although it exceeds the definition of mental health rehabilitation since the use of psychotherapeutic rehabilitation was very rare in migrant origin samples and only very limited analyses could be conducted.

### 2.5. Survey Measurements

The Hopkins Symptom Checklist-25 (HSCL-25) [32] was administered during the health examination to measure clinically significant affective symptoms in a self-administered questionnaire (some participants were interviewed because of their difficulties in reading). It includes questions on 15 depressive and 10 anxiety symptom items experienced during the past seven days. The scale ranges from 1 (“not at all”) to 4 (“extremely”). Responses were summed and divided by the number of answered items to generate a symptom mean score ranging from 1.0 to 4.0. Participants were included in the analysis if they had responded to at least 20 out of the total 25 items. The cut-off point of 1.75 was used to indicate clinically significant symptoms and was used as a dichotomous variable in the present study.

Self-reported use of health services due to mental health problems was assessed with the question “have you used any health services because of mental health problems in Finland during the past 12 months?” and perceived need for mental health services was assessed with the question “do you think you are currently in need of health services due to mental health problems?”, both with response options “yes” or “no”. Both were asked in the full interview and the former was asked also in the short interview.

### 2.6. Analyses of the Register-Based and Survey-Based Variables

We calculated the prevalence for each of the eight register-based variables. In addition, register-based and survey-based data were analyzed together in the following three ways: (1) the proportion of those receiving mental health services based on any of the register-based mental health variables (the combination variable) out of those who manifested clinically significant affective symptoms measured with the HSCL-25 (survey-based variable), (2) the proportion of those who had used general rehabilitation services among the following three groups: out of those who had purchased prescribed psychotropic medication (register-based variable), out of those who had received mental health services based on any of the register-based mental health variables (the combination variable), and out of those who reported the need for health services due to mental health problems (survey-based variable), and (3) the proportion of those reporting use of health services due to mental health problems (12 months; survey-based variable) or need of health services due to mental health problems (survey-based variable) out of those who had purchased psychotropic medication (register-based variable) or out of those receiving mental health services based on any of the register-based mental health variables (the combination variable). Since the time window for the survey questions was narrower (one year for service use) than for the registers (several years), equivalence between the two information sources was not of interest per se, but instead, possible differences between the groups that may indicate differences in under-reporting in the survey setting.

### 2.7. Statistical Analysis

Inverse probability weights (IPW) were used in all analyses in order to enhance the generalizability of the findings. The weights were calculated separately for the register sample and for those that participated in the survey. In the register sample weights, the different unequal sampling probabilities were accounted for, while logistic regression modeling was additionally used for the survey sample weights to reduce the non-response bias [33]. The differences between groups were analyzed with logistic regression, assessing the stratified sampling in the variance estimation using the Taylor linearization method. The *p*-values were based on Satterthwaite-adjusted F-values. In addition, finite population correction was applied in the analyses, because a relatively high proportion of the total population (migrant origin groups) was included in the study [34]. The predictive margins method was used to calculate model-adjusted proportions and their confidence intervals [35]. The analyses on the use of psychotherapy rehabilitation were conducted without adjustments for age or gender due to a low number of cases, as well as some other analyses in the Russian and Somali origin groups (indicated in the tables). Any *p*-values < 0.05 were considered statistically significant. All analyses were performed using SAS 9.4 and SUDAAN 11.0.1 software versions.

### 2.8. Ethical Approval

All subjects gave their informed consent for inclusion before they participated in the study. The study was conducted in accordance with the Declaration of Helsinki, and the protocol was approved by the Ethics Committee of the Coordinating Ethics Committee of the Helsinki and Uusimaa Hospital District, Finland (Maamu Study reference number: 325/13/03/00/09 and Health 2011 Survey reference number: 45/13/03/00/11).

## 3. Results

The number of participants by each used variable, gender, and age distribution of the study population are presented in Table 1. In the study sample, there were fewer men in the Russian origin group and more in the Kurdish origin group than in the general population. The Somali and Kurdish origin groups were on average younger than the general population group, in both men and women.

Register-based information on mental health-related use of health services is presented in Table 2.

Having at least one visit to hospital inpatient or outpatient care with a psychiatric diagnosis was more prevalent among the Kurdish origin group but less prevalent among the Russian and Somali origin group in comparison with the general population in Finland. When examined by gender, the differences remained statistically significant among Russian women and Somali men and women compared to the general population. Similar group differences were observed in visits to hospital inpatient or outpatient care in psychiatry and in sick leaves due to a psychiatric diagnosis when both genders were examined jointly. When examined by gender, the differences remained statistically significant among women and Somali origin men for both variables and among Kurdish origin men for care in psychiatry. Among Russian and Somali origin groups, both men and women, the purchase of prescribed psychotropic medication was less prevalent but no difference appeared between Kurdish adults and the general population. We found no difference between the groups of migrant origin and the general population in the prevalence of entitlement to reimbursement of psychotropic medicine expenses at a special rate. The results of the use of psychotherapeutic rehabilitation (without adjustments for age or gender due to a low number of cases) indicated that the proportion of those who had used psychotherapeutic rehabilitation was zero or very close to zero in all of the migrant origin groups, whereas the proportion was 1% in the general population (0.3% among men and 2% among women). According to the combination variable, including all of the six above mentioned variables, the prevalence of records on mental health-related use of health services was higher in the Kurdish origin group, men in particular, and lower in Russian and Somali origin groups, both men and women, in comparison to the general population. All migrant origin groups had used general rehabilitation services less than in the general population, women in particular.

Table 3 shows the proportion of those with a record of mental health-related use of health services (register-based information) out of those who manifested clinically significant affective symptoms (survey-based information).

Among persons exhibiting affective symptoms, the proportion of those receiving services was overall lower in Russian, Somali, and Kurdish origin groups compared with the general population. When examined by gender, the differences remained mainly statistically significant among women. The number of persons who had received rehabilitation was too low in Russian and Somali origin groups to perform all the analyses. Furthermore, too few men of Somali origin had affective symptoms to perform the analysis.

Table 4 shows the proportion of those who had received rehabilitation services (register-based information) out of those who had purchased prescribed psychotropic medication (register-based information), had record of mental health-related use of health services (register-based information, the combination variable), or reported being in mental health-related need of health services (survey-based information).

Among persons who had purchased psychotropic medication or had a record of mental health-related use of health services, the proportion of those who had used rehabilitation services was lower in Russian, Somali, and Kurdish origin groups than in the general population. When examined by gender, the differences remained mainly statistically significant among women. Among those who reported being in mental health-related need of health services, a comparison was possible only between the Kurdish origin group and the general population. The proportion of those receiving rehabilitation services was lower than in the general population. However, also in the Russian origin group, the non-adjusted analyses indicated low prevalence.

Table 5 shows the proportion of persons, who reported mental health-related use of health services (12 months; survey-based information) or self-perceived mental health-related need of health services (survey-based information) out of those, who had purchased prescribed psychotropic medication (register-based information) or having a record of mental health-related use of health services (register-based information, combination variable).

Among persons who had purchased psychotropic medication, the proportion of those reporting mental health-related use of health services was lower in the Somali origin group than in the general population, and the proportion of those reporting mental health-related need of health services was higher among the Kurdish origin group than among the general population. The results were similar when we investigated those having any register-based record on mental health-related use of health services. When examined by gender, the differences remained statistically significant among women.

## 4. Discussion

The present study explored register-based mental health-related use of health services in three migrant origin populations and the correspondence between the need for services observed in the survey and used services observed in the registers. The results indicated several interesting findings. The group of Kurdish origin differed in the use of services from the other groups of migrant origin: the people of Kurdish origin had more often psychiatric diagnoses, been treated in psychiatry more often, and had more sick leaves due to a psychiatric diagnosis in comparison with the general population. The Russian and Somali origin groups had less often psychiatric diagnoses, were less often treated in psychiatry, had less sick leave due to psychiatric diagnosis, and purchased less prescribed psychotropic medication than the general population. These findings are in line with the previous findings that the Kurdish origin adults manifest considerably more often mental health problems in comparison to the general population in Finland [17] and carry a lot of potentially traumatic burden [18]. The findings regarding the Russian and Somali origin groups were in line with some previous observations that the incidence and prevalence of mental disorders were overall lower among migrants compared with the native Finnish population when only register-based information was used [19].

All three migrant origin groups were underrepresented in the rehabilitation services of the Social Insurance Institution. Whereas 3% of the general population had used rehabilitation services during the four-year time period under the scope, the respective figures were 1.4%, 0.9%, and 0.8% for the Kurdish, Somali, and Russian origin groups. Although the findings regarding psychotherapy rehabilitation in this study are limited due to the small number of cases, the proportion of those in psychotherapy rehabilitation services was 0% in the Somali and Kurdish origin groups and 0.2% in the Russian origin group, whereas it was 1.1% for the general population and 2% for the general population women. Thus, access to rehabilitation seems to be less available for all of the migrant origin groups compared with the general population.

The correspondence between the need for services and use of services was investigated since the usage of services is important to estimate not only as such, but also taking the estimated need into account. This was possible by combining both register-based and survey data. Among those exhibiting clinically significant affective symptoms at the moment of the survey (2010–2012), 42% of the general population had at least once visited hospital inpatient or outpatient care with a psychiatric diagnosis, and 43% had at least one visit to hospital inpatient or outpatient care in psychiatry (2009–2012). The respective figures were lower in all three migrant origin groups, ranging from 11% (Russian origin group) to 18% and 23% (Kurdish origin group). This treatment gap was especially evident among women in all three groups. Among those having affective symptoms, 11% of the general population had received rehabilitation whereas the respective figure was only 3% for the Kurdish origin group and too low for the Russian and Somali origin groups to be analyzed. All these results point out that when affective symptoms are taken into account, all the three migrant origin groups are underrepresented in mental health and rehabilitation services in comparison to the general population.

The correspondence between having a record of mental health-related use of health services and receiving rehabilitation services was investigated since rehabilitation may be considered a more remedial type of treatment compared with only having a diagnosis or sick leave or lighter treatment such as psychotropic medication. Among those who had a register-based record of mental health-related use of health services, i.e., visits to hospital inpatient or outpatient care in psychiatry with a psychiatric diagnosis, purchase or entitlement to reimbursement of psychotropic medication, sick leave due to a psychiatric diagnosis, or psychotherapeutic rehabilitation, 11% of the general population had been in rehabilitation. The respective figures were lower, only 3–4% for the migrant origin groups. In the general population, 27% of those, who reported a mental health-related need for health services were receiving rehabilitation, whereas the respective figure was only 10% for the Kurdish origin group. These observations add to the previous ones regarding the access to rehabilitation being less available for migrant populations. Previous observations in Finland have also been made where non-native Finnish speakers seek Social Insurance Institution’s rehabilitation less often and positive rehabilitation decisions are less frequently admitted in comparison to native Finnish counterparts [36]. The underuse has also been observed in Finland regarding services of mental health clinics and rehabilitation services, especially for men [37].

Many of the differences observed between the migrant origin groups and the general population in mental health-related use of health services were present for both men and women. Thus, being underrepresented in mental health and rehabilitation services needs to be taken into account for both genders with a migration background. Some of the differences were more pronounced among women. This might reflect the fact that in the general population in Finland men use health services for mental health problems much less than women, e.g., in the present study only 0.3% of the general population men had used psychotherapy, in contrast to 2% of the women. One potential reason for the more noticeable unmet need of services among women might be that men, who more frequently enter the country with a refugee status and for instance with experiences of torture, end up in services more easily when in need of services, than women.

Although the migrant groups were not compared to each other, they differed from the general population to different directions in some of the register-based treatment variables. This might be considered surprising since both Somali and Kurdish origin groups were initially considered potentially vulnerable because of the common history of involuntary migration, a large amount of potentially traumatic experiences in the former home country [18], and high prevalence of perceived discrimination in the host country [15]. It is, however, in line with the previous findings from Finland that affective symptoms are highly prevalent in the Kurdish origin population in Finland and much less in the Somali population [17]. Kankaanpää [38] has suggested that besides differing health views, the low use of mental health services among Somali origin persons can indicate unfamiliarity with psychiatric services. Moreover, causal attributions of mental health problems among Somali origin populations may differ from that expected by practitioners in Finland [39]. Similar to the present study, Laban et al. [40] found that among Iraqi asylum seekers in the Netherlands, the use of mental health services was low compared to the prevalence of psychiatric disorders. Shishehgar et al. [41] outlined in their integrative review a lack of information about healthcare services as one of the factors influencing the mental health of Iranian immigrants. Based on the results of the present study, this is something to improve also in the context of Finland.

Interestingly, although the prevalence of affective symptoms of Russian origin women in Finland has been observed to be as high as 25% compared to the 10% among women in the general population, their mental health-related use of health services was much lower than that of the general population. It has been shown that the Russian origin population in Finland uses a lot of cross-border health care [42], which may explain some of the results. However, the mental health needs of Russian women may easily be overlooked as they may not receive as much attention as populations with a refugee background, whose potential vulnerability is more familiar to the service providers. This discrepancy between current affective symptoms versus the usage of services needs more attention.

The differences between the migrant origin groups in comparison to the general population were investigated in reporting the mental health-related use or need of health services in the survey among those receiving mental health-related health services based on the register data. Since the time window for the survey questions was narrower (one year for service use) than for the registers (from three to four years), equivalence between the two information sources was not of interest per se, but instead possible differences between the groups that may indicate differences in under-reporting in the survey setting. The results indicated that among those having a record of mental health-related use of health services, Kurdish origin adults reported a mental health-related need for health services more often (39%) than the general population. Thus, Kurdish origin adults who receive mental health-related health services report also more often the need for those services in the survey compared to the general population (23%). Somali origin migrants, on the contrary, reported less often mental health-related use (11%) or need (7%) of health services in the survey in comparison to the general population (33% and 23%). There might be several reasons for this finding. Firstly, it might be that either the topic of mental health is too sensitive to be revealed in the interview, especially to an interviewer who shares the same ethnic background. Secondly, it might be that the treatment itself and the reasons behind a medication do not become clear to the patient, thus a person is not aware of having a psychiatric diagnosis or being treated with psychotropic medication. These results are important for both the clinicians to improve their communication and researchers to evaluate the reliability of their survey-based findings.

From a policy perspective, it is important to distinguish and discuss the causes of the unmet need [13]. The lack of service coordination and cultural competence of the health professionals is an important barrier to acknowledge and needs improvement [43,44,45,46,47,48]. In their study of perceived need for mental healthcare among Turkish and Moroccan labor migrants in the Netherlands, Fassaert et al. [49] found that the most important barrier to care was the preference to solve the problem on one’s own, probably relating to stigma. Byrow et al. [5] also discuss concerns about confidentiality, which may be related to a general fear of authority figures, lack of trust in an interpreter who may be a part of the clients extended social network, or concern that details, which could compromise the safety of other family members living in the country of origin, will be shared. Lack of financial resources is also needed to be considered even in a Nordic welfare state model.

Perceived discrimination is also a known potential barrier to receiving services. There is significant evidence of the pervasiveness of discrimination in the Finnish society. For example, the Being Black in the EU report [50] found the rates of racist harassment to be the highest in Finland as compared to the other studied EU countries: 63% of the respondents of African descent living in Finland reported experiences of racist harassment. Experiences of discrimination have been shown to increase the odds of poor mental health among the foreign-born population in Finland [15,16].

The recent work of Salami et al. [7] suggests that strategies to improve mental health service delivery should include developing community-based services, addressing financial barriers, training immigrant service providers on mental health, increasing collaboration across sectors in mental health service delivery, and advancing the role of interpreters and cultural brokers. To address the mental health of Somali communities, Mölsä and colleagues [51] have called for culturally appropriate general and mental health services, underlining the importance of acknowledging clients’ preferences, needs, and alternative healing practices. Similarly, Pavlish et al. [6] underlined that in order to provide high quality, transcultural healthcare, providers must encourage patients to voice their own health explanations, expectations, and worries.

### Strengths and Limitations

The present study used population-based data on Russian, Somali, and Kurdish origin people in Finland. Representative data for the general population was used as a comparison. The main strengths of this study are the population-based study design, approach of combining survey-based and register-based data sources, analyzing the foreign-born population groups separately, including comparative data from the general population, and the relatively high participation rate.

This study measured affective symptoms using the HSCL-25 [32]. The cut-off point of 1.75 was applied as an indication of clinically significant symptoms equivalent to an anxiety or depressive disorder [52]. The HSCL-25 has been shown to have good reliability and validity cross-culturally [53] and in clinical refugee samples [54]. On the one hand, HSCL-25 has frequently been used to assess depression and anxiety in refugee populations [55]. On the other hand, Kuittinen and colleagues [56] demonstrated that the HSCL-25 has limitations that affect their valid use among the Russian, Somali, and Kurdish origin populations. Using the Finnish general population as a reference group may also be questioned, since the levels of mental health problems in Finland, in general, are known to be relatively high. Another limitation is that the survey and register-based data date back to 2012, thus improvements might have happened in the mental healthcare sector since then. This calls for a need to investigate the situation also with more recent data sets. Some of the results of the present study might be influenced by differing background factors between the groups, such as a higher unemployment rate in the Somali origin group, influencing e.g., the variable of sick leaves.

## 5. Conclusions

The public welfare services should adjust to the increased diversity of the population in Finland. There is no consistent national policy in Finland on organizing or improving mental health services for refugees or persons coming from similar conditions, and regional differences occur in how the special features of supporting this target group’s mental health needs have been addressed and implemented in the services [57].

The present study points out that especially when affective symptomology is taken into account, all the three migrant origin groups are underrepresented in mental health and rehabilitation services in comparison to the general population. Both the high prevalence of mental health symptoms and the underuse of mental health services call for actions to promote mental health and improve the service paths of the studied population groups. Such health promotion and service development entail several levels and involvement of different actors.

## Figures and Tables

**Table 1:** Descriptive statistics of the study population.

	Total				Men				Women			
	Russian	Somali	Kurdish	General population	Russian	Somali	Kurdish	General population	Russian	Somali	Kurdish	General population
Participants by variables, *n*												
Register-based variables	1998	1963	1948	2275	767	942	1129	1112	1231	1021	819	1163
Affective symptoms (HSCL) *	465	377	509	870	167	155	274	373	298	222	235	497
Self-reported mental health-related use of health services **	689	478	613	932	252	216	235	409	437	262	288	523
Self-reported mental health-related need for health services ***	524	331	508	1081	186	151	278	451	338	180	230	630
Gender, % (95% CI) ****												
Men	37.8 (35.6–40.0)	48.0 (46.3–49.7)	57.9 (56.7–59.1)	48.8 (46.7–50.8)								
*p*-value *****	**<0.001**	0.544	**<0.001**									
Age, % (95% CI) ****												
18–29	28.7 (26.7–30.8)	40.1 (38.4–41.7)	42.2 (41.0–43.3)	30.4 (28.5–32.3)	32.7 (29.3–36.2)	40.2 (37.8–42.6)	43.8 (42.3–45.4)	31.0 (28.3–33.8)	26.2 (23.8–28.9)	40.0 (37.7–42.3)	39.9 (38.1–41.7)	29.8 (27.2–32.5)
30–44	33.0 (30.9–35.2)	40.0 (38.3–41.7)	37.3 (36.2–38.4)	30.1 (28.2–32.0)	32.1 (288–35.6)	39.9 (37.4–42.3)	35.5 (34.0–37.0)	30.0 (27.4–32.8)	33.5 (30.8–36.3)	40.2 (37.9–42.5)	39.7 (37.9–41.5)	30.2 (27.6–32.9)
45–64	38.3 (36.1–40.6)	19.9 (18.6–21.3)	20.6 (19.6–21.5)	39.5 (37.5–41.5)	35.2 (31.8–38.8)	20.0 (18.0–22.1)	20.7 (19.4–21.9)	39.0 (36.1–41.9)	40.3 (37.4–43.1)	19.9 (18.0–21.8)	20.4 (19.0–21.9)	40.0 (37.3–42.9)
*p*-value *****	0.104	**<0.001**	**<0.001**		0.151	**<0.001**	**<0.001**		0.31	**<0.001**	**<0.001**	

* Participants of the health examination; ** Participants of the full and short interview; *** Participants of the full interview; **** Register-based information; ***** Difference compared with the reference group of general population (Satterthwaite adjusted F-statistic), bolded *p*-values represent statistically significant differences.

**Table 2:** Register-based information on mental health-related use of health services, % (95% CI).

	Total *						
	Russian (Rus)	*p*-Value, Rus vs. Gen	Somali (Som)	*p*-Value, Som vs. Gen	Kurdish (Kur)	*p*-Value, Kur vs. Gen	General Population (Gen)
Visits to hospital in-/outpatient care with a psychiatric diagnosis (2009–2012)	4.2 (3.4–5.3)	**<0.001**	3.4 (2.8–4.1)	**<0.001**	8.3 (7.6–8.9)	**0.043**	6.9 (6.0–8.1)
Visits to hospital in-/outpatient care in psychiatry (2009–2012)	3.7 (3.0–4.7)	**<0.001**	2.8 (2.3–3.5)	**<0.001**	9.7 (9.0–10.5)	**<0.001**	6.5 (5.5–7.6)
Purchase of psychotropic medication (2009–2011)	15.1 (13.6–16.7)	**<0.001**	11.9 (10.8–13.1)	**<0.001**	22.4 (21.3–23.4)	0.081	20.6 (19.0–22.3)
Reimbursement of psychotropic medication at a special rate (2009–2011)	1.0 (0.6–1.6)	0.573	1.4 (1.1–1.9)	0.525	1.3 (1.1–1.6)	0.702	1.2 (0.8–1.8)
>10 days of sick leave due to a psychiatric diagnosis (2009–2011)	0.9 (0.6–1.4)	**<0.001**	0.4 (0.2–0.6)	**<0.001**	3.7 (3.2–4.2)	**0.004**	2.4 (1.8–3.1)
Psychotherapeutic rehabilitation (2009–2012)	0.2 (NA) ***		0 (NA) ***		0 (NA) ***		1.1 (0.8–1.7)
Any register-based record on mental health-related use of health services ****	16.3 (14.8–18.1)	**<0.001**	13.7 (12.5–14.9)	**<0.001**	25.6 (24.5–26.7)	**0.004**	22.6 (20.9–24.3)
General rehabilitation (2009–2012)	0.8 (0.5–1.3)	**<0.001**	0.9 (0.6–1.2)	**<0.001**	1.4 (1.1–1.7)	**<0.001**	3.0 (2.4–3.8)
	Men **						
Visits to hospital in-/outpatient care with a psychiatric diagnosis (2009–2012)	3.9 (2.7–5.6)	0.059	3.8 (2.9–5.0)	**0.017**	7.2 (6.5–8.1)	0.117	5.9 (4.7–7.4)
Visits to hospital in-/outpatient care in psychiatry (2009–2012)	3.3 (2.2–4.9)	0.067	3.3 (2.5–4.4)	**0.027**	7.4 (6.7–8.3)	**0.008**	5.1 (3.9–6.6)
Purchase of psychotropic medication (2009–2011)	11.3 (9.2–13.7)	**0.002**	12.0 (10.4–13.8)	**0.002**	18.5 (17.2–19.8)	0.084	16.2 (14.3–18.4)
Entitlement to reimbursement of psychotropic medication at a special rate (2009–2011)	1.1 (0.6–2.1)	0.835	2.0 (1.4–2.9)	0.140	1.5 (1.2–1.9)	0.394	1.2 (0.7–2.0)
>10 days of sick leave due to a psychiatric diagnosis (2009–2011)	0.2 (NA) ***		0.5 (0.3–0.9)	**0.018**	2.0 (1.6–2.4)	0.157	1.3 (0.8–2.2)
Psychotherapeutic rehabilitation (2009–2012)	0 (NA) ***		0 (NA) ***		0 (NA) ***		0.3 (NA) ***
Any register-based record on mental health-related use of health services ****	12.5 (10.3–15.1)	**0.002**	13.9 (12.2–15.8)	**0.006**	21.4 (20.1–22.8)	**0.009**	17.9 (15.8–20.2)
General rehabilitation (2009–2012)	0.4 (NA) ***		1.1 (0.7–1.8)	0.794	1.2 (0.9–1.6)	0.920	1.2 (0.7–2.1)
	Women **						
Visits to hospital in-/outpatient care with a psychiatric diagnosis (2009–2012)	4.6 (3.5–6.0)	**0.002**	3.0 (2.4–3.8)	**<0.001**	9.3 (8.4–10.4)	0.146	7.9 (6.5–9.6)
Visits to hospital in-/outpatient care in psychiatry (2009–2012)	4.2 (3.1–5.5)	**<0.001**	2.4 (1.8–3.2)	**<0.001**	12.2 (11.1–13.4)	**<0.001**	7.7 (6.3–9.5)
Purchase of psychotropic medication (2009–2011)	18.4 (16.2–20.7)	**<0.001**	11.8 (10.4–13.4)	**<0.001**	26.1 (24.5–27.8)	0.346	24.7 (22.3–27.2)
Entitlement to reimbursement of psychotropic medication at a special rate (2009–2011)	0.9 (0.5–1.7)	0.500	0.9 (0.5–1.5)	0.429	1.1 (0.8–1.6)	0.720	1.2 (0.7–2.1)
>10 days of sick leave due to a psychiatric diagnosis (2009–2011)	1.4 (0.9–2.2)	**0.001**	0.2 (NA) ***		5.3 (4.5–6.1)	**0.011**	3.4 (2.5–4.6)
Psychotherapeutic rehabilitation (2009–2012)	0.3 (NA) ***		0 (NA) ***		0.1 (NA) ***		2.0 (1.3–3.0)
Any register-based record on mental health-related use of health services ****	19.7 (17.5–22.1)	**<0.001**	13.4 (11.9–15.0)	**<0.001**	29.5 (27.9–31.3)	0.099	26.9 (24.4–29.5)
General rehabilitation (2009–2012)	1.2 (0.7–1.9)	**<0.001**	0.6 (0.4–1.0)	**<0.001**	1.4 (1.0–1.9)	**<0.001**	4.6 (3.6–6.0)

* Adjusted by age and gender; ** Adjusted by age; *** Non-adjusted prevalence, not enough data for group comparison; **** Visits to hospital in-/outpatient care with a psychiatric diagnosis (2009–2012), visits to hospital in-/outpatient care in psychiatry (2009–2012), purchase of psychotropic medication (2009–2011), entitlement to reimbursement of psychotropic medication at a special rate (2009–2011), >10 days of sick leave due to a psychiatric diagnosis (2009–2011), or psychotherapeutic rehabilitation (2009–2012).

**Table 3:** Proportion of those with a record of mental health-related use of health services (register-based information) out of those who manifested affective symptoms (survey-based information), % (95% CI).

	Total *						
	Russian (Rus)	*p*-Value, Rus vs. Gen	Somali (Som)	*p*-Value, Som vs. Gen	Kurdish (Kur)	*p*-Value, Kur vs. Gen	General Population (Gen)
Visits to hospital in-/outpatient care with a psychiatric diagnosis (2009–2012)	10.7 (4.8–22.3)	**0.002**	14.7 (6.5–29.9)	**0.028**	17.8 (12.9–23.9)	**0.008**	41.8 (24.5–61.3)
Visits to hospital in-/outpatient care in psychiatry (2009–2012)	11.2 (5.5–21.5)	**0.001**	11.6 (4.6–26.3)	**0.012**	23.1 (17.7–29.6)	**0.040**	42.6 (25.2–62.0)
Purchase of prescribed psychotropic medication (2009–2011)	36.9 (25.3–50.3)	**0.013**	23.3 (10.9–42.9)	**0.003**	42.2 (35.6–49.1)	**0.018**	66.0 (47.0–81.0)
Any register-based record on mental health-related use of health services *****	39.8 (28.0–52.9)	**0.023**	30.5 (16.4–49.6)	**0.012**	46.9 (40.1–53.8)	0.056	66.4 (47.1–81.3)
General rehabilitation (2009–2012)	2.4 (NA) ***		NA ****		2.7 (1.2–6.0)	**0.021**	10.8 (5.0–21.5)
	Men **						
Visits to hospital in-/outpatient care with a psychiatric diagnosis (2009–2012)	10.9 (1.7–46.8)	0.137	NA ****		16.5 (9.5–27.3)	0.101	38.5 (16.6–66.3)
Visits to hospital in-/outpatient care in psychiatry (2009–2012)	16.5 (NA) ***		NA ****		21.3 (13.3–32.3)	0.246	37.7 (15.8–66.1)
Purchase of prescribed psychotropic medication (2009–2011)	58.6 (27.8–83.9)	0.698	NA ****		39.0 (28.2–51.0)	0.346	50.9 (30.1–71.4)
Any register-based record on mental health-related use of health services *****	58.7 (28.3–83.6)	0.743	NA ****		43.9 (32.7–55.7)	0.525	52.2 (30.6–73.0)
	Women **						
Visits to hospital in-/outpatient care with a psychiatric diagnosis (2009–2012)	11.0 (4.5–24.4)	**0.008**	14.2 (5.5–32.2)	**0.049**	18.2 (12.5–25.7)	**0.023**	43.8 (22.3–68.0)
Visits to hospital in-/outpatient care in psychiatry (2009–2012)	9.9 (NA) ***		10.2 (NA) ***		23.2 (16.7–31.2)	**0.034**	48.5 (26.0–71.6)
Purchase of prescribed psychotropic medication (2009–2011)	31.8 (20.6–45.7)	**0.006**	24.3 (10.2–47.6)	**0.006**	43.8 (35.7–52.2)	**0.029**	75.1 (47.4–90.9)
Any register-based record on mental health-related use of health services *****	35.6 (23.9–49.4)	**0.012**	34.5 (17.3–57.0)	**0.029**	48.2 (39.8–56.7)	0.065	75.0 (46.6–91.2)

* Adjusted by age and gender; ** Adjusted by age; *** Non-adjusted prevalence, not enough data for group comparison; **** Not enough data to perform the analysis; ***** Visits to hospital in-/outpatient care with a psychiatric diagnosis (2009–2012), visits to hospital in-/outpatient care in psychiatry (2009–2012), purchase of psychotropic medication (2009–2011), entitlement to reimbursement of psychotropic medication at a special rate (2009–2011), >10 days of sick leave due to a psychiatric diagnosis (2009–2011), or psychotherapeutic rehabilitation (2009–2012).

**Table 4:** Proportion of those who had used rehabilitation services (register-based information) out of those who had purchased psychotropic medication (register-based information), had a record of mental health-related use of health services (register-based), or reported mental health-related need of health services (survey-based information), % (95% CI).

	Total *						
	Russian (Rus)	*p*-Value, Rus vs. Gen	Somali (Som)	*p*-Value, Som vs. Gen	Kurdish (Kur)	*p*-Value, Kur vs. Gen	General Population (Gen)
Purchase of psychotropic medication (2009–2011)	2.7 (1.5–4.9)	**<0.001**	3.2 (1.8–5.4)	**<0.001**	3.8 (3.0–4.9)	**<0.001**	10.1 (7.7–13.1)
Any register-based record on mental health-related use of health services *****	3.4 (2.1–5.7)	**<0.001**	3.5 (2.2–5.7)	**<0.001**	3.7 (2.9–4.7)	**<0.001**	10.8 (8.5–13.8)
Self-reported need of health services due to mental health problems	2.5 (NA) ***		NA ****		10.0 (5.1–18.8)	**0.006**	27.3 (18.5–38.3)
	Men **						
Purchase of psychotropic medication (2009–2011)	1.8 (NA) ***		4.9 (2.5–9.3)	0.709	3.9 (2.8–5.5)	0.291	5.8 (3.1–10.4)
Any register-based record on mental health-related use of health services *****	2.6 (NA) ***		5.3 (2.8–9.5)	0.965	3.6 (2.6–4.9)	0.258	5.4 (2.9–9.6)
	Women **						
Purchase of psychotropic medication (2009–2011)	3.2 (1.6–6.3)	**<0.001**	1.3 (0.6–2.8)	**<0.001**	3.4 (2.4–4.9)	**<0.001**	13.0 (9.6–17.4)
Any register-based record on mental health-related use of health services *****	4.1 (2.3–7.1)	**<0.001**	1.7 (NA) ***		3.5 (2.5–5.0)	**<0.001**	14.5 (11.1–18.8)

* Adjusted by age and gender; ** Adjusted by age; *** Non-adjusted prevalence, not enough data for group comparison; **** Not enough cases to perform the analysis; ***** Visits to hospital in-/outpatient care with a psychiatric diagnosis (2009–2012), visits to hospital in-/outpatient care in psychiatry (2009–2012), purchase of psychotropic medication (2009–2011), entitlement to reimbursement of psychotropic medication at a special rate (2009–2011), >10 days of sick leave due to a psychiatric diagnosis (2009–2011), or psychotherapeutic rehabilitation (2009–2012).

**Table 5:** Proportion of those reporting mental health-related use or need of health services (survey-based information) out of those having purchased psychotropic medication or having record of mental health-related use of health service (register-based information % (95% CI).

	Total *						
	Russian (Rus)	*p*-Value, Rus vs. Gen	Somali (Som)	*p*-Value, Som vs. Gen	Kurdish (Kur)	*p*-Value, Kur vs. Gen	General Population (Gen)
**Out of those having purchased psychotropic medication**							
Self-reported mental health-related use of health services	22.9 (16.0–31.7)	0.050	10.6 (5.2–20.4)	**0.001**	40.1 (32.6–48.1)	0.244	33.7 (27.1–41.0)
Self-reported mental health-related need for health services	29.8 (21.0–40.4)	0.198	NA ***		36.8 (28.7–45.8)	**0.008**	22.7 (17.4–29.0)
Out of those having any register-based record on mental health-related use of health services ****							
Self-reported mental health-related use of health services	22.7 (16.1–31.1)	0.059	10.5 (5.4–19.4)	**<0.001**	39.5 (32.4–46.9)	0.204	32.8 (26.3–40.0)
Self-reported mental health-related need for health services	30.1 (21.5–40.3)	0.190	6.7 (2.0–19.5)	**0.025**	38.8 (30.9–47.3)	**0.002**	23.0 (17.9–29.1)
	Men **						
**Out of those having purchased psychotropic medication**							
Self-reported mental health-related use of health services	21.1 (10.8–37.2)	0.328	NA ***		30.3 (20.1–43.0)	>0.999	30.4 (19.8–43.5)
Self-reported mental health-related need for health services	19.7 (8.7–38.9)	0.760	NA ***		22.2 (12.6–35.9)	0.512	17.0 (9.4–28.6)
Out of those having any register-based record on mental health-related use of health services ****							
Self-reported mental health-related use of health services	20.9 (11.1–35.9)	0.324	NA ***		28.6 (19.1–40.4)	0.881	29.9 (19.3–43.1)
Self-reported mental health-related need for health services	18.1 (8.0–36.0)	0.817	NA ***		25.7 (15.9–38.7)	0.218	16.2 (9.0–27.3)
	Women **						
**Out of those having purchased psychotropic medication**							
Self-reported mental health-related use of health services	23.7 (15.4–34.7)	0.078	10.3 (4.1–23.5)	**0.004**	47.8 (37.8–57.9)	0.074	35.6 (27.8–44.3)
Self-reported mental health-related need for health services	35.8 (24.3–49.3)	0.150	NA ***		46.5 (35.3–58.1)	**0.003**	25.5 (18.9–33.4)
Out of those having any register-based record on mental health-related use of health services ****							
Self-reported mental health-related use of health services	23.5 (15.4–34.0)	0.086	10.6 (4.7–22.4)	**0.003**	47.5 (38.2–57.0)	0.050	34.7 (26.9–43.4)
Self-reported mental health-related need for health services	36.5 (25.1–49.6)	0.159	NA ***		46.7 (35.9–57.7)	**0.003**	26.6 (20.2–34.2)

* Adjusted by age and gender** Adjusted by age; *** Not enough cases to perform the analysis; **** Visits to hospital in-/outpatient care with a psychiatric diagnosis (2009–2012), visits to hospital in-/outpatient care in psychiatry (2009–2012), purchase of psychotropic medication (2009–2011), entitlement to reimbursement of psychotropic medication at a special rate (2009–2011), >10 days of sick leave due to a psychiatric diagnosis (2009–2011), or psychotherapeutic rehabilitation (2009–2012).

## References

[1] Wittchen H.-U., Jacobi F., Rehm J., Gustavsson A., Svensson M., Jönsson B., Olesen J.D., Allgulander C., Alonso J., Faravelli C. (2011). The size and burden of mental disorders and other disorders of the brain in Europe 2010. Eur. Neuropsychopharmacol..

[2] Abubakar I., Aldridge R.W., Devakumar D., Orcutt M., Burns R., Barreto M.L., Dhavan P., Fouad F.M., Groce N., Guo Y. (2018). The UCL-Lancet Commission on Migration and Health: The health of a world on the move. Lancet.

[3] Close C., Kouvonen A., Bosqui T., Patel K., O’Reilly D., Donnelly M. (2016). The mental health and wellbeing of first generation migrants: A systematic-narrative review of reviews. Glob. Health.

[4] Missinne S., Bracke P. (2010). Depressive symptoms among immigrants and ethnic minorities: A population based study in 23 European countries. Soc. Psychiatr. Psychiatr. Epidemiol..

[5] Byrow Y., Pajak R., McMahon T., Rajouria A., Nickerson A. (2019). Barriers to Mental Health Help-Seeking Amongst Refugee Men. Int. J. Environ. Res. Public Health.

[6] Pavlish C., Noor S., Brandt J. (2010). Somali immigrant women and the American health care system: Discordant beliefs, divergent expectations, and silent worries. Soc. Sci. Med..

[7] Salami B., Salma J., Hegadoren K. (2018). Access and utilization of mental health services for immigrants and refugees: Perspectives of immigrant service providers. Int. J. Ment. Health Nurs..

[8] Gilliver S.C., Sundquist J., Li X., Sundquist K. (2014). Recent research on the mental health of immigrants to Sweden: A literature review. Eur. J. Public Health.

[9] Lindert J., Von Ehrenstein O.S., Priebe S., Mielck A., Brähler E. (2009). Depression and anxiety in labor migrants and refugees—A systematic review and meta-analysis. Soc. Sci. Med..

[10] Norredam M.L., García-López A., Keiding N., Krasnik A. (2009). Risk of mental disorders in refugees and native Danes: A register-based retrospective cohort study. Soc. Psychiatr. Psychiatr. Epidemiol..

[11] Miliband D., Tessema M.T. (2018). The unmet needs of refugees and internally displaced people. Lancet.

[12] Fazel M., Wheeler J., Danesh J. (2005). Prevalence of serious mental disorder in 7000 refugees resettled in western countries: A systematic review. Lancet.

[13] Allin S., Masseria C. (2009). Unmet need as an indicator of health care access. Eurohealth.

[14] Guidi C., Malmusi D., Palencia L., Ferrini S. (2015). Inequalities by immigrant status in unmet needs for healthcare in Europe. Eur. J. Public Health.

[15] Rask S., Elo I.T., Koskinen S., Lilja E., Koponen P., Castaneda A.E. (2018). The association between discrimination and health: Findings on Russian, Somali and Kurdish origin populations in Finland. Eur. J. Public Health.

[16] Castaneda A.E., Rask S., Koponen P., Suvisaari J., Koskinen S., Härkänen T., Mannila S., Laitinen K., Jukarainen P., Jasinskaja-Lahti I. (2015). The Association between Discrimination and Psychological and Social Well-being. Psychol. Dev. Soc..

[17] Rask S., Suvisaari J., Koskinen S., Koponen P., Mölsä M., Lehtisalo R., Schubert C., Pakaslahti A., Castaneda A.E. (2015). The ethnic gap in mental health: A population-based study of Russian, Somali and Kurdish origin migrants in Finland. Scand. J. Public Health.

[18] Castañeda A., Junna L., Lilja E., Skogberg N., Kuusio H., Mäki-Opas J., Koponen P., Suvisaari J. (2017). The prevalence of potentially traumatic pre-migration experiences: A population-based study of Russian, Somali and Kurdish origin migrants in Finland. J. Trauma. Stress Disord. Treat..

[19] Markkula N., Lehti V., Gissler M., Suvisaari J.M. (2017). Incidence and prevalence of mental disorders among immigrants and native Finns: A register-based study. Soc. Psychiatr. Psychiatr. Epidemiol..

[20] Kieseppä V., Torniainen-Holm M., Jokela M., Suvisaari J., Gissler M., Markkula N., Lehti V. (2019). Immigrants’ mental health service use compared to that of native Finns: A register study. Soc. Psychiatr. Psychiatr. Epidemiol..

[21] Bhopal R.S. (2013). Migration, Ethnicity, Race, and Health in Multicultural Societies.

[22] Rechel B., Mladovsky P., Devillé W. (2012). Monitoring migrant health in Europe: A narrative review of data collection practices. Health Policy.

[23] Alang S.M. (2015). Sociodemographic disparities associated with perceived causes of unmet need for mental health care. Psychiatr. Rehabil. J..

[24] Official Statistics of Finland (OSF) Population Structure [e-publication]. ISSN=1797-5395. Annual Review 2019. Helsinki: Statistics Finland. http://www.stat.fi/til/vaerak/2019/02/vaerak_2019_02_2020-05-29_tie_001_en.html.

[25] European Migration Network Key Figures on Immigration 2019. Finnish Immigration Service, 2020. http://www.emn.fi/files/2024/EMN_tilastokatsaus_2019_EN_naytto.pdf.

[26] Castaneda A.E., Rask S., Juntunen T., Skogberg N., Tolonen H., Koskinen S. (2018). Enhancing survey participation among foreign-born populations: Experiences from the Finnish migrant health and wellbeing study (Maamu). Finn. Yearb. Popul. Res..

[27] Koskinen S., Lundqvist A., Ristiluoma N. (2012). Health, Functional Capacity and Welfare in Finland in 2011.

[28] ICD-10 Version: 2010. https://icd.who.int/browse10/2010/en#/V.

[29] WHO Collaborating Centre for Drug Statistics Methodology ATC/DDD Codes. Norwegian Institute of Public Health: Oslo, Norway. http://www.whocc.no/atc_ddd_index/.

[30] Social Insurance Institution Vaikeat Psykoosit Ja Muut Vaikeat Mielenterveyden Häiriöt. Entitlements for Reimbursement at a Special Rate: 112 Severe Psychoses and Other Severe Psychiatric Disorders..

[31] Social Insurance Institution What is Rehabilitation?. https://www.kela.fi/web/en/what-is-rehabilitation.

[32] Derogatis L.R., Lipman R.S., Rickels K., Uhlenhuth E.H., Covi L. (1974). The Hopkins Symptom Checklist (HSCL): A self-report symptom inventory. Syst. Res. Behav. Sci..

[33] Robins J.M., Rotnitzky A., Zhao L.P. (1994). Estimation of regression coefficients when some regressors are not always observed. J. Am. Stat. Assoc..

[34] Lehtonen R., Pahkinen E.J. (2004). Practical Methods for Design and Analysis of Complex Surveys.

[35] Graubard B.I., Korn E.L. (1999). Predictive margins with survey data. Biometrics.

[36] Härkäpää K., Vuorento M., Buchert U., Lehikoinen T. (2012). Maahanmuuttajat Kelan kuntoutuspalveluissa. Kuntoutusta hakeneet, kuntoutuspäätökset ja myönnetyt toimenpiteet. Kuntoutus.

[37] Gissler M., Malin M., Matveinen P. (2006). Terveydenhuollon palvelut ja sosiaalihuollon laitospalvelut. Maahanmuuttajat ja Julkiset Palvelut.

[38] Kankaanpää S. (2018). Mental Health among Somali Origin Migrants in Finland: Considerations for Depressive Symptom Manifestation, Causal Attributions of Mental Health Problems, and Psychiatric Assessment.

[39] Kuittinen S., Mölsä M., Punamäki R.-L., Tiilikainen M., Honkasalo M.-L. (2017). Causal attributions of mental health problems and depressive symptoms among older Somali refugees in Finland. Transcult. Psychiatr..

[40] Laban C.J., Gernaat H.B.P.E., Komproe I.H., De Jong J.T.V.M. (2007). Prevalence and predictors of health service use among Iraqi asylum seekers in the Netherlands. Soc. Psychiatr. Psychiatr. Epidemiol..

[41] Shishehgar S., Gholizadeh L., Digiacomo M., Green A., Currow D.C. (2016). Health and socio-cultural experiences of refugee women: An integrative review. J. Immigr. Minor. Heal..

[42] Kemppainen L., Kemppainen T., Skogberg N., Kuusio H., Koponen P. (2017). Immigrants’ use of health care in their country of origin: The role of social integration, discrimination and the parallel use of health care systems. Scand. J. Caring Sci..

[43] Mills S., Xiao A.Q., Wolitzky-Taylor K., Lim R., Lu F.G. (2017). Training on the DSM-5 cultural formulation interview improves cultural competence in general psychiatry residents: A pilot study. Transcult. Psychiatr..

[44] Aggarwal N.K., Lam P., Castillo E.G., Weiss M.G., Diaz E., Alarcón R.D., Van Dijk R., Rohlof H., Ndetei D.M., Scalco M. (2015). How do clinicians prefer cultural competence training? Findings from the DSM-5 cultural formulation interview field trial. Acad. Psychiatr..

[45] Adeponle A.B., Thombs B.D., Groleau D., Jarvis E., Kirmayer L.J. (2012). Using the cultural formulation to resolve uncertainty in diagnoses of psychosis among ethnoculturally diverse patients. Psychiatr. Serv..

[46] Diaz E., Añez L.M., Silva M., Paris M., Davidson L. (2017). Using the cultural formulation interview to build culturally sensitive services. Psychiatr. Serv..

[47] Kirmayer L.J., Groleau D., Guzder J., Blake C., Jarvis E. (2003). Cultural consultation: A model of mental health service for multicultural societies. Can. J. Psychiatr..

[48] Lewis-Fernández R., Aggarwal N.K., Bäärnhielm S., Rohlof H., Kirmayer L.J., Weiss M.G., Jadhav S., Hinton L., Alarcón R.D., Bhugra D. (2014). Culture and psychiatric evaluation: Operationalizing cultural formulation for DSM-5. Psychiatry.

[49] Fassaert T., De Wit M.A.S., Tuinebreijer W.C., Verhoeff A.P., Beekman A.T.F., Dekker J. (2008). Perceived need for mental health care among non-western labour migrants. Soc. Psychiatr. Psychiatr. Epidemiol..

[50] (2018). Second European Union Minorities and Discrimination Survey. Hum. Rights Doc. Online.

[51] Mölsä M., Kuittinen S., Tiilikainen M., Honkasalo M.-L., Punamäki R.-L. (2016). Mental health among older refugees: The role of trauma, discrimination, and religiousness. Aging Ment. Heal..

[52] Nettelbladt P., Hansson L., Stefansson C.-G., Borgquist L., Nordström G. (1993). Test characteristics of the Hopkins Symptom Check List-25 (HSCL-25) in Sweden, using the Present State Examination (PSE-9) as a caseness criterion. Soc. Psychiatr. Psychiatr. Epidemiol..

[53] Bean T., Derluyn I., Eurelings-Bontekoe E., Broekaert E., Spinhoven P. (2007). Validation of the multiple language versions of the Hopkins Symptom Checklist-37 for refugee adolescents. Adolescence.

[54] Hollifield M., Warner T.D., Lian N., Krakow B., Jenkins J.H., Kesler J., Stevenson J., Westermeyer J. (2002). Measuring trauma and health status in refugees. JAMA.

[55] Bogić M., Njoku A., Priebe S. (2015). Long-Term mental health of war-refugees: A systematic literature review. BMC Int. Health Hum. Rights.

[56] Kuittinen S., Velázquez R.G., Castaneda A.E., Punamäki R.-L., Rask S., Suvisaari J. (2016). Construct validity of the HSCL-25 and SCL-90-Somatization scales among Russian, Somali and Kurdish origin migrants in Finland. Int. J. Cult. Ment. Health.

[57] Castaneda A.E., Mäki-Opas J., Jokela S., Kivi N., Lähteenmäki M., Miettinen T., Nieminen S., Santalahti P. (2018). Paloma Expert Group. Supporting Refugees’ Mental Health in Finland. PALOMA Handbook.

